# Diabetes management in Guinea Bissau: a situational analysis

**DOI:** 10.11604/pamj.2019.34.10.19874

**Published:** 2019-09-04

**Authors:** Jorge César Correia, Adalgisa Lopes, Cumba Bispo Iala, N’Boma Adriano Sanca, Augusto Bidonga, Grégoire Lagger, Alain Golay, Montserrat Perolini Castellsague

**Affiliations:** 1 Division of Tropical and Humanitarian Medicine and Division of Therapeutic Patient Education for Chronic Diseases, Department of First Aid Medicine, WHO Collaboration Center, Geneva University Hospitals, Geneva, Switzerland; 2 Swiss Association for Aid to Diabetic People in Guinea-Bissau, Geneva, Switzerland; 3 Department of Internal Medicine, Simão Mendes National Hospital, Avenida Francisco Mendes Bissau, Guinea-Bissau; 4 Department of Intensive Care Medicine, Simão Mendes National Hospital, Avenida Francisco Mendes Bissau - Guinea-Bissau; 5 Service of Pediatrics, Renato Grandi Foundation, Guinea-Bissau; 6 Division of Therapeutic Patient Education for Chronic Diseases, Department of First Aid Medicine, WHO Collaboration Center, Geneva University Hospitals, Geneva, Switzerland; 7 Division of Therapeutic Patient Education for Chronic Diseases, Department of First Aid Medicine, WHO Collaboration Center, Geneva University Hospitals, Geneva, Switzerland; 8 Division of Tropical and Humanitarian Medicine, Geneva University Hospitals, Geneva Switzerland

**Keywords:** Diabetes care, Guinea-Bissau, health system organization, chronic disease

## Abstract

**Introduction:**

There is an increasing commitment in the African Region towards diabetes care, following acknowledgement that it is an important public health issue which needs to be addressed in order to improve population health. We conducted a situational analysis of diabetes care in Guinea Bissau in order to identify the main issues faced in the management of the disease in this country.

**Methods:**

The study design was qualitative and data collection was done using semi directive interviews and focus groups with participants involved in primary diabetes care and management in Guinea Bissau (health care professionals, non-governmental organization staff, traditional healers) and patients. The data was analyzed using the five-phase approach of the thematic analysis framework.

**Results:**

The major themes identified included: the lack of specialists and properly trained healthcare personnel; no standardized care protocol for diagnosis, treatment, follow up and proper management for diabetic patients; resources poor primary health care settings; no validated epidemiological dataset on prevalence and the lack of awareness about diabetes (in general population and also in medical staff).

**Conclusion:**

This first situational analysis can serve as a baseline to develop an action plan to address the main issues identified.

## Introduction

A fast change in the demographic status, the socio-cultural lifestyle as well as financial transitions have become the drivers for rising numbers of diabetes mellitus (DM) cases within the sub-Saharan African region [1,2]. As a result, increasingly diabetes and associated complications are the underlying cause of premature deaths and disability [3,4]. That is the case of Guinea Bissau, a country characterized by a resource poor health system [5]. There is an increasing commitment in the country towards diabetes care, following acknowledgement that it is an important public health issue which needs to be addressed in order to improve population health. Optimal diabetes management requires an organized, systematic approach and the involvement of a coordinated team of dedicated health care professionals working in an environment where patient-centered high-quality care is a priority [6,7]. In order to improve patient care, it is important to conduct an accurate needs assessment and identification of the priority areas that must be improved [8,9]. Therefore, we decided to conduct a situational analysis of diabetes care in Guinea Bissau with the aim of determining what factors need to be addressed before planning and designing an effective intervention. This research study was developed based on the work experience of the researchers who wish to design an evidence-based intervention which will be viable in the particular, resource and manpower poor setting of health care in Guinea Bissau.

## Methods

**Participant selection and sampling:** prior to the start of the interviews with the relevant health care professionals (HCP), the General Secretary of the Ministry of Foreign Affairs and International Cooperation and the General Director of Health Institutions from the Ministry of Health were involved in the initial discussion of the goals of the study and which sites were to be selected in order to get the best picture of the situation of diabetes care in the country. We decided to interview HCP involved in DM care from different levels of the healthcare system: one referral hospital in the capital, the Hospital Nacional Simão Mendes (HNSM) and two rural health care centers, namely the Cacheu and Pelundo primary healthcare centers. We also sought to interview traditional healers since in Guinea Bissau, particularly in rural areas, the general population use their services. We wanted to evaluate whether traditional healers have the needed awareness and skills to provide adequate treatment and management for diabetic patients who opt to use their services. Only one traditional healer accepted to be interviewed. Furthermore, the staff from several NGOs involved in diabetes care were also included in our investigation which were previously identified by the research team and further informed by the interviews with the HCP. The following NGOs were selected for the study: “Ayuda, Intercambio y Desarrollo” (AIDA), “Ceu e Terra”(CT), the “Associação nacional de defesa dos pacientes diabeticos” (ANDPD) and “Associação de luta contra a diabetes em Guinée Bissau” (ASLUCO Diabetes GB). In order to identify the entire spectrum of challenges encountered in the management of this disease we also interviewed the diabetic patients themselves and let them put forth their difficulties and views on possible solutions. These patients were recruited through ASLUCO Diabetes-GB, a local diabetic patient run NGO. Twenty patients responded to our call. Participants of all ages came with type 1 diabetes (3 children with their mother) and type 2 diabetes (17 adults). We used a heterogeneous purposive sampling approach [10] when recruiting the participants during the visits of the different selected sites. The inclusion criteria for the participants included all those involved in the care of DM affected patients and willing to participate in the study. The characteristics of the different participants are presented in Table 1.

**Table 1:** Characteristics of the participants interviewed

Structure type	Affiliation	Position
Government	Ministry of health	General Director of Health Institutions
	Ministry of health	Social worker
	Ministry of Foreign Affairs and International Cooperation	General Secretary
Tertiary hospital	HNSM	General Director
	HNSM	Clinical Director
	HNSM	Deputy Clinical Director
	HNSM	Administrator SMNH
	HNSM	Director of Human Resources
	HNSM	Head of Emergency care
	HNSM	Head of pediatrics
	HNSM	Head of orthopedic surgery
	HNSM	Head of intensive care
	HNSM	Physician in paediatrics
	HNSM	Physician in ggynaecology
	HNSM	Head nurse in gynaecology
	HNSM	Nutritionist
Rural health care centers	Cacheu primary healthcare center	Clinical director
	Pelundo primary healthcare center	Head nurse
Non-Governmental Organizations (NGO)	AIDA	Logistics manager
	AIDA	General Manager
	ANDPD	President
	ANDPD	Attending physician
	ANDPD	Administrator
	ANDPD	Medical student and health educator
	CT	Pediatrician
	CT	Head nurse
	ASLUCO Diabetes GB	President
	ASLUCO Diabetes GB	Nurse
Traditional Healer’s home where he receives patients	NA	NA

**Data collection:** the participants were initially contacted through a call or an email in which the research team gave a full explanation on the objectives of this study'>when recruiting the participants during the visits of the different selected sites. The inclusion criteria for the participants included all those involved in the care of DM affected patients and willing to participate in the study. The characteristics of the different participants are presented in Table 1.

**Data collection:** the participants were initially contacted through a call or an email in which the research team gave a full explanation on the objectives of this study, the mission as well as the methodology selected for data collection. This initial contact gave all the required information on the study to the participants. Regarding the perspectives of the HCP the mission as well as the methodology selected for data collection. This initial contact gave all the required information on the study to the participants. Regarding the perspectives of the HCP, traditional healer and NGO staff traditional healer and NGO staff, the data was collected by conducting semi directive interviews using a specifically designed open-ended interview guide (Annex 1). Each of the interviews was done in the workplace or office of the participant. Regarding the perspectives of the diabetic patients we conducted a focus group. After an introduction, the research team explained the purpose of the activity. The patients were asked to introduce themselves in a few words (name, type of diabetes and treatments with the circumstance of discovery). Once the presentations were done, we encouraged them to express themselves using the photolanguage technique [11]: patients chose pictures cut out of newspapers beforehand. The instruction was to choose an image that speaks to them in connection with their disease. Then we let the patients express themselves freely through the chosen picture which allowed us to reflect on their experience, their knowledge of the disease and difficulties. Throughout the interviews and patient focus group, the interviewers made efforts to clarify information without influencing or contributing with any views (so as to avoid bias and confounding in the data collection process). The interviewers made immediate written notes of the data after the interviews and focus group in order to ensure that none of the details were omitted. All three interviewers compared their notes, a means to validate the collected data. As part of the ethical aspects of the study, oral consent was obtained from all those who agreed to participate in the study and the workshops.

**Annex 1:**
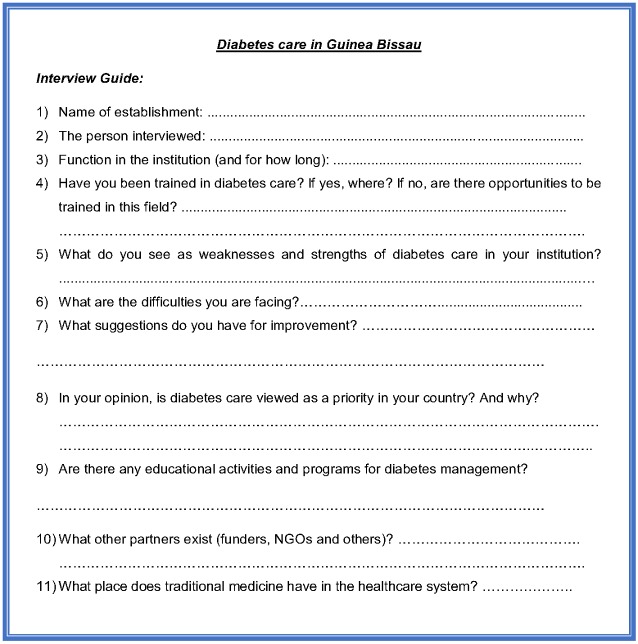
Semi directive open-ended questionnaire which served as the interview guide

**Data analysis:** the documented data was analyzed by applying thematic analysis involving a comparative strategy to the interview results as well as use of an iterative process for identifying similarities as well as variations in each of the transcripts [12]. In phase 1, the data was read again and again as an iterative process so as to become familiar with it, notes were made and sections of potential interest to the research objectives were highlighted [13]. In phase 2, using the constructs and framework of the constructive grounded theory, coding was done. This consisted of open coding wherein the first examination of the data was done and key phrases of relevance to the research objectives were identified. Axial coding was done to classify the identified codes as per their related features. In phase 3 of the thematic analysis, the codes were used to determine the key recurrent and emerging themes or main concepts [14]. The data from the workshop and patient focus group conducted was used in the coding stages to validate the emerging themes. In phase 4, a review of the possible themes was done to differentiate them from simple codes. In phase 5, the themes were named and phase 6 of the thematic analysis consisted of reporting/writing the results.

## Results

**Figure 1:**
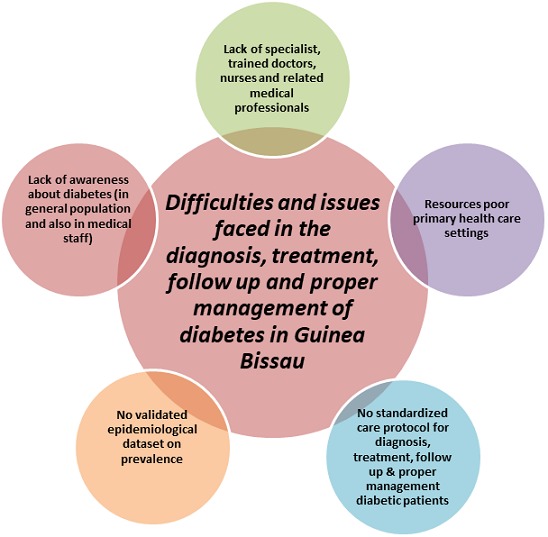
Identified issues faced in the treatment and management of diabetes in Guinea Bissau

**Key themes identified:** the main themes of relevance which were identified include: lack of specialists, trained doctors, nurses and related medical professionals; resources poor primary health care system; no standardized care protocol for diagnosis, treatment, follow up and proper management of the disease; no validated epidemiological dataset on prevalence; and lack of awareness about diabetes in general population and also in medical staff (Figure 1). All the themes tend to overlap and are interlinked. Thus, each issue has a detrimental impact on the other issues and ultimately result in the poor quality of diabetes care that is prevalent in Guinea Bissau today.

**Lack of specialists, trained doctors, nurses and related medical professionals:** all of the participants confirmed that there are no endocrinologists in the country and that diabetes care was exclusively provided by general physicians not properly trained in the management of the disease. The most common diabetic complication seen is the diabetic foot and there are no specially trained physicians or wound care nurses to provide proper care, leading to high levels of amputation rates which is associated with high levels of mortality. In addition, there are not sufficient numbers of trained nutritionists to educate and counsel diabetic patients on dietary requirements. There is also a severe lack of psychologists to provide patients help to cope with the burden of this disease. Many people still use the services of traditional healers who have no awareness of diabetes and its management and only refer the patients to the health sector when complications arise.

**Resources poor primary health care settings:** the hospitals and NGOs lack funding to provide the much-needed screening for detecting people at risk or at an early stage of the disease before complications arise. There is a lack of diagnostic devices like glucometers and patients have to buy treatment essentials such as diagnostic strips, bandages and medication, even insulin. Oftentimes medication runs out in the country and patients are forced to procure it in the neighboring countries. Furthermore, there are not enough healthcare professionals which results in a very high work load, combined with lack of training results in poor care, little to no monitoring of glucose levels and onset of avoidable complications. The doctors and other staff are very poorly paid so there is no motivation to provide better care. The lack of funding also affects the patient nutrition since the nutritionists are unable to make diabetic friendly meals for the inpatients.

**No standardized care protocol for diagnosis, treatment, follow up and proper management for diabetic patients:** since the health care system has no standardized diabetes care protocol and cases are managed by single general practitioners, the primary care provided is based on whatever the attending doctor decides to do. Since no standardized, multi-disciplinary team is there to care for diabetics, there is often no coordination between attending general physicians, the orthopedic department (who attends to patients suffering from the diabetic foot complications) and the nursing staff and nutritionists.

**No validated epidemiological dataset on prevalence:** the disease burden and actual prevalence of this public health issue is difficult to estimate due to a lack of large-scale epidemiological studies on this issue in Guinea Bissau. The current knowledge of diabetes in Guinea Bissau is based largely on epidemiological data taken during sporadic community testing initiatives conducted by certain NGOs and hospital data from patients who present with diabetes complications.

**Lack of awareness about diabetes:** most patients come to know of their disease only when complications like diabetic foot and other issues like cardiovascular complications set in. The pediatric and pregnant cases of diabetes have no awareness of how to manage the disease and lack of diagnostics as well as food uncertainty makes it difficult to treat the disease. There is also an issue of follow up which is not possible for patients from rural areas since this follow up is provided by NGOs that are not accessible to them.

## Discussion

Diabetes is becoming an increasing problem in Guinea Bissau [15,16].The data collected indicates that diabetes treatment, management and follow up are quite poor in spite of the fact that non-communicable diseases have been attended to within the Health Strategic Plan of Guinea Bissau [17]. The most important challenge identified was the severe lack of resources in this poor country. Hospitals, especially in primary care facilities, lack basic diagnostic tools such as glucose meters, testing strips, and laboratory capacity to measure glycosylated hemoglobin (HbA1C). This is consistent with several studies in African countries [18-20]. In addition, health care expenditure is mostly out-of-pocket. Patients, including children and pregnant women have to pay directly for their medication and care materials (p.e test strips and bandages) on their own. This makes correct treatment and management impossible due to the low economic status of the population, further propagating the vicious cycle of poverty as medicines consume large portions of household incomes [21-23]. As a result, the diabetic population of this nation has a very high prevalence of diabetes-related complications in particular foot complications. Furthermore, there is a lack of properly trained health workforce. This study found that most of the attending physicians are trained for general medicine and do not have the experience needed to provide proper diabetes management. That is the case of many African countries where the emphasis and focus on training health care providers about diabetes was often sacrificed in the interest of promoting education for heavily funded diseases including TB, HIV, and malaria [21,24].

We further identified a lack of established protocols or guidelines for the diagnosis, treatment, management and follow up of diabetic patients. These guidelines could provide much needed guidance to LMIC clinicians. However, as in most sub-Saharan African countries, guidelines continue to rely heavily upon the guidelines used to govern the care of diabetes in high income countries like the United States, including guidelines such as the American Diabetes Association (ADA) [21]. Most of the participating health professionals stated that they provide care and services to diabetic patients using their own views and often without any multidisciplinary approach which is essential for the correct treatment and management of diabetes mellitus and its complications. Regarding patient education and self-management of the disease, a proven effective strategy in diabetes care, it is practically inexistent in Guinea Bissau. The rare programs that exist are provided by NGOs due to lack of trained and qualified personnel in hospitals. Several studies conducted in sub-Saharan Africa demonstrated the feasibility and effectiveness of patient self-management education programs but remain under-developed and do not constitute a priority in these resource poor settings [25]. The data from participants of this study has also highlighted the issue of lack of awareness in the general population about diabetes. Some awareness campaigns are being run by the NGOs using media like the radio. Once again, due to lack of resources, the campaigns are few and rural populations may not have access to them. The situation is further aggravated by the fact that many continue to use services of traditional healers who are unaware of diabetes. Most of them end up in hospitals with severe diabetic complications which could have been avoided if there was more awareness.

## Conclusion

Our findings suggest that any intervention that is designed in the future has to take the identified themes into account and find suitable solutions that can work in Guinea Bissau. In addition, further research is needed to find prevalence data which is at present not very comprehensive due to lack of epidemiological studies. The Guinea Bissau health care system requires funds, training for specialized medical staff and educational campaigns directed towards diabetes awareness. This will help in improving the present situation of poor diabetes management in the country.

### What is known about this topic

- Diabetes care requires a patient centered multi-disciplinary team approach;
- There is a lack of reliable evidence about the true magnitude of the burden of disease and its complications in LMICs, and what interventions will or will not be effective in this context;
- As diabetes prevalence increases in LMICs, there is a need for more evidence to guide health systems organisation and planning.

### What this study adds

- This is the first analysis of diabetes care in Guinea Bissau;
- The health system in Guinea Bissau is struggling to provide basic care responding to diabetic patients needs and expectations;
- Our results can serve as a baseline to develop an action plan to address the main issues identified.

## Competing interests

The authors declare no competing interest.
